# Occupational accident fatalities in Brazil: A time series study from 2011 to 2021

**DOI:** 10.1371/journal.pone.0321550

**Published:** 2025-04-16

**Authors:** Ramon Evangelista dos Anjos Paiva, Thiffany Nayara Bento de Morais, Ketyllem Tayanne da Silva Costa, Renan Cipriano Moioli, Angelo Giuseppe Roncalli da Costa Oliveira, Fábia Barbosa de Andrade

**Affiliations:** 1 Doctor in Public Health, Department of Public Health, Federal University of Rio Grande do Norte, Brazil,; 2 Department of Nursing, Federal University of Rio Grande do Norte, Brazil,; 3 Bioinformatics Multidisciplinary Environment, Digital Metropolis Institute, Federal University of Rio Grande do Norte, Brazil,; 4 Doctor of Social Dentistry, Department of Public Health, Federal University of Rio Grande do Norte, Brazil,; 5 Doctor of Health Science, Department of Nursing, Federal University of Paraíba, Brazil; Arak University of Medical Sciences, IRAN, ISLAMIC REPUBLIC OF

## Abstract

In Brazil, accidents at work generate temporary or permanent disability or death, with 2,556 deaths in 2021. The objective of this work is to evaluate the lethality rate due to formal work accidents in relation to the Brazilian regions, in the historical series of 2011 and 2021. An ecological time series analysis using secondary data from reports of accidents at work available from the National Institute of Social Security. To calculate the lethality rates, we used occupational accidents and mortality by region in Brazil. Brazil presented a rate of 61 deaths for every 100,000 formal work accidents in 2011, the country presents a downward curve to 32 deaths for 100,000 work accidents in 2011, being a decrease of 52% in relation to the beginning. The world panel shows how much Brazil is part of a scenario seen around the planet, with 318,000 deaths due to accidents and 2 million due to work-related diseases. Accidents at work account for 18% of deaths in low- and middle-income countries such as Brazil, compared to only 5% in high-income countries. This study revealed the need to strengthen public policies such as the National Workers’ Health Policy, including the strengthening of Renast or even a more robust intersectoral approach.

## Introduction

According to the International Labor Organization, illness and accidents in the workplace can now be considered a silent global epidemic, with 140 million new cases every year and 2.4 million work-related accidents and deaths every year. Despite knowledge of the causes and consequences of accidents at work, these figures don’t reveal the real seriousness of the situation, since there are a large number of informal workers who are not registered [1].

An accident at work, conceptually referred to as a typical accident at work, can be characterized as occurring in the course of the insured person’s work, where they suffer bodily injury or a functional disorder that causes death, loss or reduction, whether permanent or temporary, of their ability to work [2]. The Reporting of Workplace Accidents must be made by the first working day after its occurrence, and in the event of death it must be issued immediately.

In this sense, it is clear that work is a determinant of the health-disease process and can have beneficial or harmful effects on the worker’s life. Lifestyle and work have a strong influence on people’s quality of life, which is why there is a need to bring about changes in the way we perceive work, in order to protect workers and their families and communities. Therefore, it is understandable that the beneficial effects are related to workers who have better conditions in their work environment, while the harmful effects are more pronounced for those who are not supported and have precarious conditions that make them more vulnerable to risks [3].

Occupational health psychology, for example, offers insights into the effects of stress, workload, and social support on workers’ mental and physical health, while the social determinants approach highlights the structural inequalities that make certain groups of workers more vulnerable. This will strengthen the fight for the right to work and live in healthy and dignified environments, while at the same time preventing social injustices and inequities [4].

According to the database of the Statistical Yearbook of Accidents at Work in Section I subsection D of the Yearbook of Brazil, the content that deals with the Communication of Accidents at Work, according to records of benefits of an accident nature granted by the National Institute of Social Security, had a total of 536,174 accidents at work recorded in 2021, an increase of approximately 15% compared to the previous year in which 465,772 accidents at work were recorded. Accidents at work can cause temporary or permanent disability or death. In 2021, there were 2,556 deaths, an increase of 19% compared to the previous year with 2,132 worker deaths, the number of permanent disabilities remained close to 5,638, and there was also a significant increase in absences from work [5].

However, in the Brazilian context, the numbers become even more significant, with a high prevalence of informal work reaching 38.6% and 25.1% working independently. Despite being significant and representative data in Brazil’s reality, the measurement of workplace accidents among this group is limited. Furthermore, these workers are likely more exposed to risks and vulnerability issues, which are of great importance to Brazil’s economic landscape, yet there are still major shortcomings in providing safety and stability. Added to this is the analysis of the living conditions this group faces, reinforcing that, in addition to heightened exposure to physical risks, there are also related psychosocial issues [6]. Thus, the study’s research question is: What is the fatality rate due to formal work accidents in Brazil?

The information above shows how necessary it is to think of strategies to deal with accidents at work. In line with this action, the World Health Organization has drawn up strategic guidelines and highlighted the need to update legislation and technical regulations on workers’ health, and for workers to have access to and coverage by health services, along with information systems, epidemiological surveillance and research into diseases, accidents and deaths at work [1]. The quest to improve work through public policies is something that goes hand in hand with the history of the Unified Health System, as in the 8th National Health Conference in 1986. Article 6 of the Organic Health Law highlights Workers’ Health as one of the lines of action of the Health Policy. The Consolidation of Labor Laws, which deals with the rules of the contractual relationship between company and employee [7].

In addition, as a way of improving progress and further consolidating a policy to promote workers’ health beyond the Consolidation of Labor Laws, in 1978 the Ministry of Labor and Employment’s Regulatory Standards were created, which are complementary provisions to Chapter V (Occupational Safety and Medicine) of Title II of the Consolidation of Labor Laws, as amended by Law No. 6,514 of December 22, 1977, and establish the minimum conditions for work environments in order to promote workers’ safety and health [8].

All these historical advances are directly related to the National Social Security Institute in Brazil, which characterizes accidents at work. It also investigates the causes of accidents. During the period in which the worker is off work, the INSS is responsible for paying benefits in cases of temporary incapacity, permanent incapacity and death, as well as providing medical assistance to the injured, their professional rehabilitation and the prevention of occupational accidents. And this is due to the integration of occupational accident insurance into social security, made by Law No. 5316 of September 14, 1967 [9].

However, it is important to emphasize that this practice, despite its great relevance, has significant economic implications for the worker, the company, and also for the State. In addition to the costs related to the accident itself, investment is needed for medical assistance and treatment for the patient, as well as expenses for hiring a new professional. Thus, even if indirectly, and often without a clear quantifiable relationship between these two factors, it is crucial to highlight that these are costs that could often be avoided, yet they represent significant economic burdens for both the private sector and the healthcare system. This underscores the importance of investing in workplace accident prevention policies.

It is unquestionable that the progress of workers’ health in Brazil was its constitutional merit as an area within the scope of public health. However, despite the criticism of its institutionalization and progress, even though it is insufficient to meet the reality of the worker and the ordinary user in Brazil, there has been countless progress in these 30 years of the SUS [10]. Therefore, the aim of this study was to assess the fatality rate caused by formal work accidents in relation to the Brazilian regions, in the historical series from 2011 to 2021.

## Materials and methods

This study is an ecological time series analysis using information from secondary sources. The research in question was conducted in Brazil and aims to evaluate the fatality rate caused by formal work accidents in relation to the Brazilian regions, in the historical series of 2011 and 2021.

Brazil is a developing country with approximately 210 million residents, comprising 26 states and the Federal District, organized into five regions—North, Northeast, Southeast, South, and Center-West—based on sociodemographic and spatial characteristics.

### Data collection

The data was obtained from May 26, 2022 to June 15, 2022, from the database of the National Institute of Social Security, of formal workers who register the Communication of Accidents at Work.

According to Article 19 of Law No. 8,213/1991, a work-related accident is defined as an accident that occurs during the performance of work on behalf of a company or a domestic employer, causing bodily injury or functional impairment that results in death, loss, or reduction (permanent or temporary) of the ability to work.

The dependent variables of the study are the number of worker deaths and accidents, while the independent variables used were region and years of the study. For inclusion criteria, the study defined that only records of formal workers would be used, the accidents must be work-related, and must have been recorded within the analyzed period. Incomplete or inconsistent data were excluded.

### Information analysis

Once the data had been collected, they were stored in Microsoft Excel®. During this stage, the databases were processed, selecting the relevant information for the research in question. Zeroed or inconsistent information was excluded, and the database was organized to meet the model requirements for Joinpoint analysis.

After this stage, Joinpoint® software was used, a program used for linear regression analysis, whose software is capable of identifying and analyzing trends in a historical series, determining whether they are stationary, ascending or descending, which uses junction point models and employs Poisson regression to estimate the Annual Percentage Change and the Average Annual Percent Change. A 5% significance level was adopted for all the trends identified [11]. A 5% significance level helps ensure that conclusions drawn from studies are more accurate and less prone to random errors, especially in public health.

Joinpoint employs a statistical method called the permutation test, which is a robust approach for assessing the significance of change points. This method involves comparing the observed time series with series generated by randomly permuting the model residuals (the differences between observed and fitted values). The test calculates the distribution of statistics of interest under the null hypothesis (no change points) and compares it with the observed model.

Joinpoint was used as it is a statistical technique designed to identify points in a time series where a significant change in trend occurs. These change points help better describe the evolution of a variable over time, fitting segmented models to assess trends over different periods.

In order to calculate the lethality rates, work accidents and mortality rates by region in Brazil were used. To do this, the following procedure was carried out: the number of deaths (O) divided by the number of accidents (A) in order to determine the lethality rate, represented by the formula: (1) Lethality = O/A = (Deaths/occupational accident) X 100,000.

### Ethical support

The information used in this research was obtained from secondary sources, specifically from publicly accessible databases, so it was not necessary to submit the study for analysis and approval by the Research Ethics Committee, as established by Brazilian Resolution No. 466, dated December 12, 2012 [12]. The authors did not have access to personal information, as the data were collected in an aggregated form, given that this was an ecological study.

## Results

Table 1 shows the demographics of the formal workers who were registered through the Communications of Accidents at Work. It can be inferred that 76.01% in Brazil are male, and this characteristic is repeated in the other regions, with the North having the highest proportion of men who suffered an accident at work at 79.15% (158395). According to the data collected, the region with the lowest rate was the Southeast, with 66.81%.

**Table 1:** Distribution of formal work accidents according to sex and marital status in Brazil between 2011 and 2021, Brazil, 2023.

Variable	Brazil	North	Northeast	Southeast	South	Midwest
	%(f)	%(f)	%(f)	%(f)	%(f)	%(f)
**GENDER**						
Male	76.01	79.15	73.30	66.81	67.72	71.39
	(5073569)	(158395)	365399	(1960173)	(745908)	(242484)
Female	23.99	20.85	26.70	33.19	32.28	28.61
	(1601210)	(41736)	(133109)	(973678)	(355501)	(97186)
**MARITAL STATUS**						
Married	37.32	27.14	36.14	39.53	35.12	33.55
	(1795975)	51505	171822	1090175	366751	115722
Divorced	7.28	5.73	4.87	7.44	8.16	7.48
	(350361)	(10878)	(23173)	(205243)	(85268)	(25799)
Unmarried	54.87	66.88	58.66	52.44	56.15	58.60
	(2640764)	(126942)	(278889)	(1446340)	(586454)	(202139)
Widowed	0.53	0.25	0.33	0.58	0.57	0.38
	(25345)	(471)	(1546)	(16074)	(5953)	(1301)

Source: National Social Security Institute, 2023.

As for marital status, widowers and divorcees had the lowest proportions, while married and single people had the highest proportions in Brazil, with more than half (54%) of accidents being suffered by single people, while married people accounted for 37.32% of accidents. When looked at by region, the North stands out as having 66.88% of accidents suffered by single people, while the Southeast has 52.44%, even below the national average.

**Fig 1:**
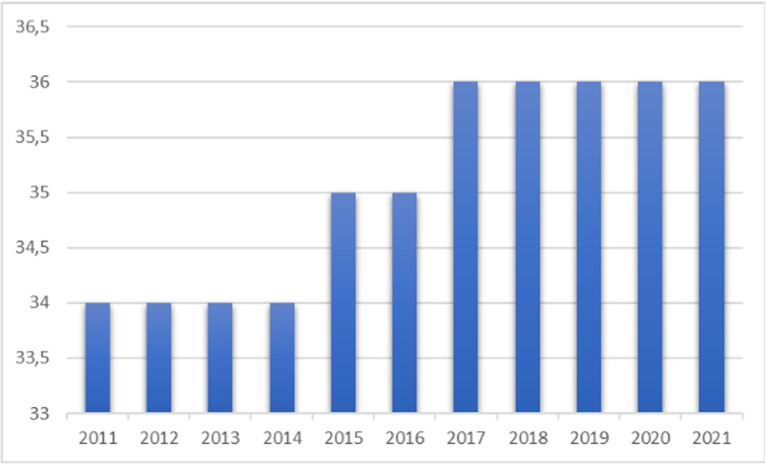
Analysis of the average age of formal workers who suffered an accident at work in Brazil between 2011 and 2021. Brazil, 2023.

Fig 1 shows the behavior of the average age of workers who have suffered an accident at work, in which the average ranged from 34 to 36 years. This variation showed a growing average over the initial period, 2011, to the end, 2021.

**Fig 2:**
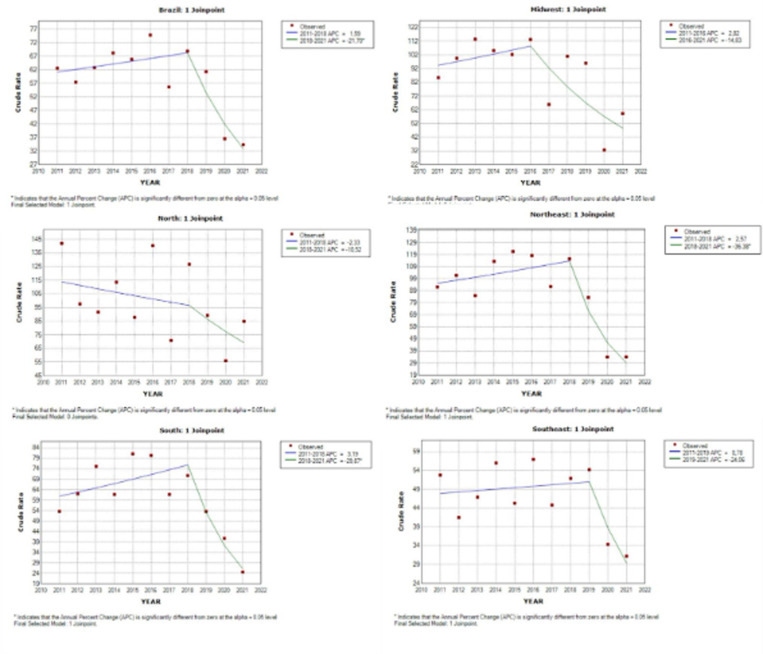
Lethality rate caused by occupational accidents in Brazil, 2011 to 2021. Brazil, 2023.

Fig 2 shows the fatality rate caused by accidents at work from 2011 to 2021 in all regions of Brazil. Brazil had 61 deaths for every 100,000 formal work accidents in 2011, Joinpoint® the country shows a growth curve to 68 worker deaths in 2018, however, in 2021 the country shows a downward curve to 32 deaths for 100,000 work accidents, a decrease of 52% compared to the beginning. The region with the highest fatality rate, the North, had the highest fatality rate among Brazilian regions, with around 113 deaths for every 100,000 occupational accidents, and this may be the reason why this curve is behaving differently from the others, where Joinpoint® has reduced this number to 96 deaths for every 100,000 occupational accidents, and if we analyze the other regions before starting a drop in mortality, they had peaks in their Joinpoint®s. In 2021, the northern region fell by around 39%, with 69 deaths for every 100,000 accidents at work.

The southern region showed the greatest growth from 2011 to the Joinpoint® mark, with around 25% growth in relation to the start of the lethality rate. The southern region had 60 deaths for every 100,000 accidents at work, and in 2018 it showed a growth curve to 75 deaths for every 100,000 accidents at work. The northeast region showed the biggest decline in the fatality rate among the other regions, starting at 95 and rising to 113 deaths per 100,000 occupational accidents and falling to 29 deaths per 100,000 occupational accidents, totaling approximately 70% less than Joinpoint® in 2018.

Although the Southeast and Midwest do not have a large variation between the lethality rate from the initial year of the analysis, 2011, to Joinpoint®, followed by the end of the analysis in 2021, it should be noted that the Midwest has a lethality rate twice as high as the Southeast, with the Midwest having a rate of 94 deaths for every 100,000 occupational accidents, while the Southeast has only half this number.

Table 2 which presents the statistical data from the linear regression of the lethality rate in Brazil and its respective regions, between 2011 and 2021, shows the presence of a Joinpoint® in Brazil in 2018, the same occurring in the North, Northeast and South regions. On the other hand, in the Southeast, there is a later Joinpoint® in 2019, while in the Midwest, where there is the first Joinpoint® in 2016, the first in the timeline. Looking at Table 2, it can be seen that the period from 2018 to 2021 showed a correction in Brazil with a p-value within the reliability parameter, which is also reproduced when the analysis looks at the whole period, as well as the Northeast and South regions.

**Table 2:** Joinpoint® analysis of the Work-related Accident Lethality rate in Brazil and its regions, 2011 to 2021. Brazil, 2023.

Local	Joinpoint®	Period	APC^1^	Lower	Upper	AAPC^2^	Lower	Upper
Brazil	2018	2011 to 2018	1,6	-4,1	7,6	-6,0*	-11,6	7,6
		2018 to 2021	-21,7*	-37,0	-2,7			
North	2018	2011 to 2018	-2,3	-13,9	10,9	-4,9	-16,8	8,8
		2018 to 2021	-10,5	-44,3	43,7			
Northeast	2018	2011 to 2018	2,6	-6,4	12,4	-11,1*	-19,3	-2,1
		2018 to 2021	-36,4*	-54,8	-10,4			
South	2018	2011 to 2018	3,2	-3,4	10,3	-8,1*	-14,3	-1,4
		2018 to 2021	-29,9*	-45,3	-10,1			
Southeast	2019	2011 to 2019	0,8	-3,8	5,5	-4,8	-11,6	2,6
		2019 to 2021	-24,1	-50,3	16,0			
Midwest	2016	2011 to 2016	2,8	-20,7	33,2	-6,4	-19,2	8,4
		2016 to 2021	-14,8	-34,3	10,4			

^1^annual percentage change; ^2^average annual percentage change.

* Indicates that the Annual Percent Change (APC) is significantly different from zero at the alpha = 0.05 level.

Source: National Social Security Institute, 2023.

## Discussion

The results of this study revealed the different profiles of the fatality rate in Brazil and its regions over the period from 2011 to 2021. In general, these results show an increase in the lethality rate of accidents at work in all regions except the North. The discussion about the impact of work is relevant in view of its relationship as a determinant of the health situation of workers, their families and the community. The results show that working in precarious conditions has negative impacts such as workers falling ill and dying. As well as having an impact on the health of workers, production processes often have an impact on the territory, including the environment and communities, and even more distant places [13].

However, the world panel shows how Brazil has more than 2 million deaths, with 318,000 deaths due to accidents and 2 million due to work-related illnesses. Accidents at work account for 18% of deaths in low- and middle-income countries such as Brazil, compared to only 5% in high-income countries [14]. According to Fig 2, which deals with the lethality rate of formal accidents at work, it is possible to understand that Fatal Accidents at Work have a significant contribution to mortality, and because they are deaths that could have been avoided, they become a significant public health problem. Therefore, records and information must be rigorous in order to adequately plan and manage this serious public health problem [15].

Every year, an average of 2.3 million people die worldwide from work-related causes. When analyzing the reality in Brazil, there are around 2,500 deaths per year on average, which is a sad statistic of one death every 3.5 hours. According to Brazil’s Social Security Secretariat, the impact on the country’s economy was around 330 million reais (101.5 million dollars) in 2016 due to accidents at work/illnesses. This figure refers only to social security benefits, excluding the associated health costs, which would make the figure even more significant [16].

As a way of mitigating the problem, the National Workers’ Health Policy (Ministerial Order/Ministry of Health No. 1. 823/2012 7) is an important tool for reducing lethality rates, with its principles, guidelines and strategies in all spheres of SUS management, federal, state and municipal, to promote workers’ health, with a focus on surveillance, reducing the damage caused by development models and production processes, in addition to the intersectoral approach that is necessary to support Workers’ Health, such as the Labor Prosecutor’s Office, Workers’ Health Surveillance, among others [17].

As for the Labor Public Prosecutor’s Office (MPT), its role is extremely important, whether in dialogue or in taking action on behalf of workers, through Conduct Adjustment Terms (TAC) in the face of precarious work situations in order to comply with current worker health and safety standards. Therefore, the Labor Prosecutor’s Office can balance the forces between worker and employee in preventing adverse conditions through its commitment to the health of workers in defense of decent work, as advocated by the International Labor Organization [17].

The Labor Prosecutor’s Office opposed the Executive Branch, declaring a total veto of the legislative bill that created the labor “reform”. In this declaration, the Labor Prosecutor’s Office states that the law will result in the end or weakening of social rights, with a reduction in workers’ legal social protection, without due social dialogue and in violation of the democratic principle of the prohibition of social retrogression [18,19].

Another recent change, Ordinance No. 217, of March 2023, amends Ministerial Office/Ministry of Health Consolidation Ordinance No. 4, of September 28, 2017, to replace the aggravation “Accident at work: serious, fatal and in children and adolescents” with “Accident at work” on the National List of Compulsory Notification of diseases, aggravations and events in public health, in public and private health services throughout the national territory, being an important milestone to speed up the competent bodies to act on the study and encourage future actions to prevent such acidentes [20].

And another front that strengthens decent work are the Regulatory Standards themselves, mentioned in Chapter V of the Consolidation of Labor Laws, created to promote safe environments for workers, helping to prevent worker death or illness, since 1978 with the publication of the first guidelines to date, adding up to 38 regulations on the most diverse professions, and new standards may emerge to adapt to the national reality [21].

In line with experiences in other countries, fatal accidents in Chile are concentrated in small and medium-sized companies, with 75% and the majority (63%) occurring in the workplace. However, one relevant piece of information is that more than half of fatal injuries occur to workers who have been with the company for less than a year, reinforcing the need for a more emphatic prevention policy with new employees and for them to be presented with all the risks to which they are subjected, and the fragility of this policy can be seen in the fact that the majority of companies that had a fatal accident in the two years of the study did not have a risk prevention department, did not have a joint occupational safety committee and some had already been fined previously, indicating a general disregard for regulatory compliance [22].

While in Italy for the period 2006–2014, there are regional differences in the average mortality rates from accidents at work and occupational accidents. Regional differences in mortality rates are inversely associated with the employment rate or GDP, and directly associated with the age of the worker. Therefore, regional differences in the average mortality rate from accidents at work in Italy for the years 2006–2014 seem to be positively correlated with the resident foreign population and inversely with GDP and the employment rate, while accidents are negatively associated with regional GDP and positively associated with the employment rate [23].

In Turkey it can be seen that even though the number of jobs is growing, the number of occupational injuries and the incidence and mortality rates are decreasing. However, when this scenario is viewed from a broader timeline, it is possible to see that the rate recorded in the occupational mortality rate/all recorded injuries is increasing. The mortality rate/all recorded injuries per 1,000 injuries increased from 8.6 in 1988 to 25.5 in 2011. For every working day, an average of five people died from occupational injuries [24].

While fatal work-related injuries in Alaska during 2004–2018, will reach 517 work-related deaths, an average of 34.5 deaths per year. The average annual risk of death over the 15-year period was 9.6 deaths per 100,000 workers, with a maximum of 13.4 in 2006 and a minimum of 5.8 in 2009. The workers who died were predominantly white (334, 71.8%), male (487, 94.2%) and residents of Alaska (362, 70.0%), with an average age of 41.7 years, ranging from 16 to 86 years [25].

A study carried out in Brazil showed that between 2008 and 2014, the South and Southeast regions had the highest incidences of accidents at work, according to Fig 2, with 17.96 and 14.28 in 2008, falling to 12.44 and 10.16, with reductions of 30.71% and 28.82%, respectively. The Midwest region showed the biggest reduction in incidence, from 12.42 to 8.31, i.e. a reduction of 31.31%. The North showed the smallest variation, going from 12.40 to 9.12, a fall of 26.47% [26].

The most economically developed regions in Brazil are those with the highest incidence of accidents at work, and these are the South and Southeast. It can therefore be seen that regions with higher levels of development need more solid strategies to deal with accidents at work, in order to prevent economic development from increasing the chances of suffering some kind of accident [27].

An accident at work is directly related to the worker’s health, while it also has a direct impact on the workplace, with the worker’s absence affecting the task he or she was performing. However, accidents at work also cause damage to the worker’s family, the company and the Social Security system, which is perceived through accident benefits, a drop in productivity and early lives lost [28]. The publication of the National Workers’ Health Policy in 2012 can be considered an important action to guide actions and scientific production in the area, as it is the normative reference for principles and guidelines in the area of Workers’ Health [4], but it cannot be the only tool for tackling or responding to the number of work-related deaths, understanding the need for other fronts in view of the complexity of dealing with this field of knowledge.

Even before the Labor Reform was approved, official statistics already showed that the country had data indicating “insufficiency in its preventive aspects related to workers’ health”: between 2010 and 2014, 3,317,932 Communications of Accidents at Work (CATs) were issued. In general terms, “an accident at work is one suffered by an employee under the employer’s responsibility, or by a special insured person, in the course of their professional activity, which causes a permanent or temporary loss or reduction in working capacity, which is equivalent to the situations provided for in Article 21 of Law No. 8,213 of 1991 [3].

Law No. 13,467 of July 13, 2017, also known as the “labor reform”, brought significant changes to the Consolidation of Labor Laws, which is the main legislation regulating labor and employment relations (2) in Brazil. According to its proposers and supporters, the aim of the labor reform was to adapt Brazilian legislation to the new labor relations that have emerged in recent decades since the Consolidation of Labor Laws was published. It was also claimed that the reason for the need to change labor legislation was to combat unemployment and the economic crisis the country was going through in 2017. The government considered that reforming labor legislation would be a way of stimulating the country’s economy by creating new Jobs [29].

The need to study the data obtained from sources related to accidents and deaths among workers aims to reduce the gaps caused by the limitations of information systems, the main consequence of which is a lack of knowledge about the impact of work on health and the absence of organized responses on the part of the SUS in relation to its prevention and control [30].

Strengthening the National Workers’ Health Policy has the effect of improving Workers’ Health Surveillance, and in this sense it is possible to reverse the situation in the world of work. This surveillance is aware of the reality of the working population and the determining factors of health problems. As a result, its actions are able to have a positive impact on workers’ health, as it is an interdisciplinary, multi-professional, inter-institutional and intersectoral practice, going beyond the limits of the health sector and bringing the analysis of the relationship between health and the work process into the context of health services [4,26].

One of the explanations for the reduction in the lethality rate may be related to the decrease in the participation rate of people in the labor market, indicating that many have given up looking for work, thus, this information justifies the reduction in records of accidents at work during the pandemic [31], and according to the annual report of the International Labor Organization (Panorama Laboral 2020) for Latin America and the Caribbean, the unemployment rate registered a significant increase in 2020.

There was an increase of 2.5 percentage points compared to the previous year, resulting in an increase of 5.4 million unemployed people, bringing the total to 30.1 million. These findings show the need to monitor cases of mortality due to accidents at work, considering the magnitude of the knowledge of the reality of each Brazilian region, in order to strengthen preventive policy and the protection of rights at work and workers’ health in Brazil.

A fatal workplace accident is often the result of intense work flows, as seen in kanban or just-in-time models, where the pace of activities exhausts time for breaks and rest. Consequently, physical, mental, and psychological fatigue can result in accidents and death [32]. This issue is among the most significant public health problems worldwide, affecting not only workers but also governments and companies, causing economic damage, widespread distress in society, and irreparable emotional and familial consequences [33,34].

The family unit suffers the most from the death of a worker, experiencing emotional disruption and financial instability. This social violence weakens the family and places it at further risk. Although the death of workers has negative impacts across various areas mentioned above, the quality of data in information systems is a real concern, given the need for accurate data to generate health promotion actions and prevent fatal accidents [35].

In addition to the fragility of data quality, there is significant underreporting, with discrepancies of up to 53% when comparing the Mortality Information System (SIM) and the Communication of Work Accidents (CAT) from the INSS. This underreporting is further compounded by informal work, accidents unrelated to service provision, data entry errors, among other factors that can elevate these rates and reveal a severe scenario of fatal work accidents, leading to potential social problems [36].

This highlights the need for reliable records to develop effective preventive policies and surveillance measures. Moreover, the authors reference qualitative and primary studies to capture the perceptions of families and work teams, which are not reached by current data collection systems. Such studies help to better understand the damage caused by fatal work accidents [37].

Drumont also points to underreporting as a prevalent bias in the notification of fatal work accidents (FWA), which are recorded in various sources of information, posing a challenge due to the absence of a unifying variable that would allow automatic cross-referencing between information systems [33].

The effects of fatal work accidents observed by families included decreased academic performance among children, impacts on health, and significant behavioral changes. For the spouse, the consequences included shattered dreams and interrupted ways of life, revealing emotional and psychological damage, as well as the presence of depression and the use of medication. These findings confirm that the psychosocial effects of a fatal work accident extend beyond the workplace and profoundly affect the victim’s family [38].

The limitations of this study concern the use of secondary data, which can be influenced by underreporting in order to effectively record illnesses and deaths from accidents at work. What would have optimized the information would have been a unification of the Information System, which would guarantee more robust data to reorient policies, planning and evaluation for workers’ health.

## Conclusion

This study carried out an analysis of the epidemiological behavior of the lethality of occupational accidents and revealed an impact in the Central-West and South regions, where there was an increasing pattern until 2012, followed by a fall until the end of the period studied, and with regard to accidents, an upward behavior in occupational accidents was identified throughout Brazil, but with a fall in the number of accidents until 2015. It can be seen that the strengthening of public policies such as the National Workers’ Health Policy, together with intersectoral inspection actions and investments in actions to prevent accidents and promote the health of workers, has led to a decline in these rates.

Furthermore, despite the decrease in the lethality of accidents at work since 2012 in Brazil, in order to reduce these numbers even further, a comprehensive approach is needed, involving actions such as compliance with safety standards, regular inspections, investigation and recording of accidents, work safety programs, improvement of working conditions, tax incentives and benefits, strengthening of the work environment, public policies, integration with the community and continuous monitoring. Only through a joint and continuous effort by employers, workers, government and society is it possible to create a safe working environment and reduce accidents.

## References

[1] Organização Mundial de Saúde. Organização Pan-Americana da Saúde (PAHO). Plano de Ação sobre a Saúde dos Trabalhadores. Tema 4.7 da agenda CD54/10, Rev.1, 1º de outubro de. 2015. Available from: https://iris.paho.org/bitstream/handle/10665.2/33985/CD54_10Rev.1-por.pdf?sequence=1&isAllowed=y

[2] Portal da Câmara dos Deputados [Internet]. Available from: https://www2.camara.leg.br/legin/fed/lei/1991/lei-8213-24-julho-1991-363650-publicacaooriginal-1-pl.html

[3] Brasil. Lei nº 8.213, de 24 de julho de 1991. Dispõe sobre os Planos de Benefícios da Previdência Social e dá outras providências. Available from: https://www2.camara.leg.br/legin/fed/lei/1991/lei-8213-24-julho-1991-363650-publicacaooriginal-1-pl.html

[4] Brasil. Portaria Federal GM/MS n° 1.823, de 23 de agosto de 2012. Institui a Política Nacional de Saúde do Trabalhador e da Trabalhadora. Diário Oficial da União. Ano CXLIX Nº 165, Seção I. p. 46-51. Brasília, 2012. Available from: https://bvsms.saude.gov.br/bvs/saudelegis/gm/2012/prt1823_23_08_2012.html

[5] Brasil. Ministério da Previdência Social. Anuário de Acidentes de Trabalho 2021. Disponível em: <https://www.gov.br/previdencia/pt-br/assuntos/previdencia-social/arquivos/onlinte-aeps-2021-/secao-iv-2013-acidentes-do-trabalho/capitulo-31-acidentes-do-trabalho>

[6] Instituto Brasileiro de Geografia e Estatística (IBGE). Pesquisa Nacional por Amostra de Domicílios Contínua Mensal [Internet]. Rio de Janeiro: IBGE; 2023 [citado em 2024 nov. 1]. Disponível em: https://www.ibge.gov.br/estatisticas/sociais/trabalho/9171-pesquisa-nacional-por-amostra-de-domicilios-continua-mensal.html

[7] Brasil. Lei 8080 de 19 de setembro de 1990. Dispõe sobre as condições para a promoção, proteção e recuperação da saúde, a organização e o funcionamento dos serviços correspondentes e dá outras providências. 1990. Available from: https://www.planalto.gov.br/ccivil_03/leis/l8080.htm#:~:text=LEI%20N%C2%BA%208.080%2C%20DE%2019%20DE%20SETEMBRO%20DE%201990.&text=Disp%C3%B5e%20sobre%20as%20condi%C3%A7%C3%B5es%20para,correspondentes%20e%20d%C3%A1%20outras%20provid%C3%AAncias.

[8] Brasil. Lei nº 6.514, de 22 de dezembro de 1977. Altera o Capítulo V do Titulo II da Consolidação das Leis do Trabalho, relativo a segurança e medicina do trabalho e dá outras providências. Disponível em: http://www.planalto.gov.br/ccivil_03/LEIS/L6514.htm

[9] Pavésio L. O papel do Instituto Nacional de Previdência Social nos acidentes do trabalho. Rev Saúde Pública [Internet]. 1973 Mar;7(1):51–61. Disponível em: doi: 10.1590/S0034-89101973000100005 4725265

[10] Gomez CM, Vasconcellos LCF de, Machado JMH. Saúde do trabalhador: aspectos históricos, avanços e desafios no Sistema Único de Saúde. Ciênc saúde coletiva [Internet]. 2018 Jun;23(6):1963–70. Disponível em: doi: 10.1590/1413-81232018236.04922018 29972503

[11] AAPC. Average Annual Percent Change (AAPC) and Confidence Interval. Surveillance Research Program. 2022. Disponível em: <https://surveillance.cancer.gov/help/Joinpoint/setting-parameters/method-and-parameters-tab/apc-aapc-tau-confidence-intervals/average-annual-percent-change-aapc>

[12] Brasil. Ministério da Saúde. Conselho Nacional de Saúde. Resolução nº 466, de 12 de dezembro de 2012. Trata sobre as diretrizes e normas regulamentadoras de pesquisas envolvendo seres humanos. Diário Oficial da União, Brasília, DF, 12 dezembro de. 2012.

[13] Bahia. Secretaria da Saúde do Estado da Bahia. Superintendência de Vigilância e Proteção da Saúde. Diretoria de Vigilância e Atenção à Saúde do Trabalhador. Centro Estadual de Referência em Saúde do Trabalhador. Guia para Análise da Situação de Saúde do Trabalhador – SUS/Bahia. Organizado por Eliane Cardoso Sales e Joselita Cássia Lopes Ramos. SESAB/ SUVISA/DIVAST/CESAT - Salvador: DIVAST, 2014

[14] Takala J, Hämäläinen P, Saarela KL, Yun LY, Manickam K, Jin TW, et al. Global estimates of the burden of injury and illness at work in 2012. J Occup Environ Hyg. 2014;11(5):326–37. doi: 10.1080/15459624.2013.863131 24219404 PMC4003859

[15] Batista AG, Santana VS, Ferrite S. Registro de dados sobre acidentes de trabalho fatais em sistemas de informação no Brasil. Ciênc saúde coletiva [Internet]. 2019 Mar;24(3):693–704. Available from: doi: 10.1590/1413-81232018243.35132016 30892492

[16] Brasil. Ministério da Previdência Social. Bases de dados históricos da Previdência Social. 2017. Acessado em 4 de julho de 2023 http://www3.dataprev.gov.br/temp/DACT01consulta32082337.htm

[17] Pereira MDS, Oliveira KT de, Silva IA. Atuação intersetorial em saúde do trabalhador. Cad Psicol Soc Trab. 2018:119–31. doi: 10.11606/issn.1981-0490.v21i2p119-131

[18] Ministério Público do Trabalho. Pedido de veto total ou parcial do Projeto de Lei da Câmara n. 38/2017. Acesso em: 1º jun. 2018 Disponível em: http://www.diap.org.br/images/stories/PLC38MPT-Pedido-de-Veto.pdf.

[19] Camargo Neto RB de. Terceirização ilícita e atuação do Ministério Público do Trabalho em face da “reforma” trabalhista. BoletimESMPU [Internet]. 30º de junho de 2019 [citado 27º de novembro de. 2023;(53):279-303. Disponível em: https://escola.mpu.mp.br/publicacoescientificas/index.php/boletim/article/view/510

[20] Brasil. Ministério da Saúde. Portaria nº 217, de 1º de março de 2023. Altera o Anexo 1 do Anexo V à Portaria de Consolidação GM/MS nº 4, de 28 de setembro de 2017, para substituir o agravo “Acidente de trabalho: grave, fatal e em crianças e adolescentes” por “Acidente de Trabalho” na Lista Nacional de Notificação Compulsória de doenças, agravos e eventos em de saúde pública, nos serviços de saúde públicos e privados em todo o território nacional.

[21] Brasil. Ministério do Trabalho e Emprego. Inspeção do Trabalho Segurança e Saúde no Trabalho. Comissão Tripartite Paritária Permanente - CTPP. Normas Regulamentadoras - NR Normas Regulamentadoras - NR. Disponível em: https://www.gov.br/trabalho-e-emprego/pt-br/assuntos/inspecao-do-trabalho/seguranca-e-saude-no-trabalho/ctpp-nrs/normas-regulamentadoras-nrs>

[22] Bachelet VC. Work-related injuries resulting in death in Chile: a cross-sectional study on 2014 and 2015 registries. BMJ Open. 2018;8(6):e020393. doi: 10.1136/bmjopen-2017-020393 29886445 PMC6009517

[23] La Torre G, Verrengia G, Saulle R, Kheiraoui F, Mannocci A. Determinantes regionais de acidentes de trabalho na Itália. Med Lav [Internet]. 28 de junho de 2017 [citado em 2 de agosto de. 2023;108(3):209-21. Disponível em: https://www.mattioli1885journals.com/index.php/lamedicinadellavoro/article/view/6127 10.23749/mdl.v108i3.6127 28660872

[24] Turkkan A, Pala K. Trends in occupational injuries and fatality in Turkey. Int J Occup Saf Ergon. 2016;22(4):457–62. doi: 10.1080/10803548.2016.1153224 27064344

[25] Lucas D, Fitzgerald E, Case S, O’Connor M, Syron L. Persistent and emerging hazards contributing to work-related fatalities in Alaska. Am J Ind Med. 2020 Aug;63(8):693–702. Epub 2020 Jun 1. Disponível em: <https://www.ncbi.nlm.nih.gov/pmc/articles/PMC8095070/ ; PMCID: PMC8095070 32483827 10.1002/ajim.23137 PMC8095070

[26] Bezerra JC, Arantes LJ, Shimizu HE, Merchán-Hamann E, Ramalho WM. Workers’ Health in Brazil: Accidents recorded by Social Security from 2008 to 2014. Rev Bras Enferm [Internet]. 2020;73(6):e20180892. Available from: doi: 10.1590/0034-7167-2018-0892 32785512

[27] Vargas FEB. O mercado de trabalho e a questão do emprego no brasil: integração precária e desenvolvimento desigual. Revista Brasileira de Sociologia. 2(4):1-24, 2014, julho-dezembro. Available from: https://rbs.sbsociologia.com.br/index.php/rbs/article/view/124/58

[28] Possebom G, Santos Alonço A. Panorama dos acidentes de trabalho no Brasil. Nucleus, Ituverava, 15(2):15-22, out. 2018. ISSN 1982-2278. Disponível em: http://nucleus.feituverava.com.br/index.php/nucleus/article/view/2691

[29] Reis JT dos, Prado AZ. reforma trabalhista brasileira de 2017 e a desconsideração da duração do trabalho como norma relacionada à saúde dos trabalhadores. Revista de Direito da Faculdade Guanambi. 2019 Jul 14;6(01):e246. Available from: https://portaldeperiodicos.animaeducacao.com.br/index.php/RDFG/article/view/13919/7681

[30] Cavalcante CAA, Cossi MS, Costa RRO, Medeiros SM, Menezes RMP. Análise crítica dos acidentes de trabalho no Brasil. Revista de Atenção à Saúde. 2015;13(44):100–9. doi: 10.13037/rbcs.vol13n44

[31] Organização Internacional do Trabalho. COVID-19 deixa um rastro de alto desemprego, inatividade e empregos precários na América Latina e no Caribe [Internet]. 2020 [cited 2023 Jun 15]. Available from: https://www.ilo.org/brasilia/noticias/WCMS_764677/lang--pt/index.htm

[32] Garcés M, Stecher A. El trabajo en tiempos de lean management: una revisión crítica sobre sus efectos adversos en las experiencias de trabajo. Innovar. 2021;31(79):61–78. doi: 10.15446/innovar.v31n79.91889

[33] Drumond E de F. Employment precariousness and the importance of the assessment of fatal occupational injuries on the Mortality Information System. Epidemiol Serv Saúde [Internet]. 2023;32(3):e2023797. Available from: doi: 10.1590/S2237-96222023000300012 37909523 PMC10615182

[34] Gonçalves Filho AP, Ramos MF. Acidente de trabalho em sistemas de produção: abordagem e prevenção. Gest Prod [Internet]. 2015;22(2):431–42. doi: 10.1590/0104-530X857-13

[35] Lacerda KM. Acidente de trabalho, precarização e desproteção social: elementos para uma discussão sobre morte e trabalho, Dissertação (Mestrado) Universidade Federal da Bahia. Salvador, 2012.

[36] Machado Ageu de Araújo. Análise da subnotificação de acidentes de trabalho fatais no Brasil. Dissertação (mestrado). Universidade Estadual de Maringá. 63f. Maringá, PR, 2021.

[37] Galdino A, Santana V, Ferrite SS. Fatores associados à qualidade de registros de acidentes de trabalho no Sistema de Informações sobre Mortalidade no Brasil. Cadernos de Saúde Pública [online]. v. 36, n. 1 [Acessado 29 Outubro 2024]. e00218318. Disponível em: ISSN 1678-4464. doi: 10.1590/0102-311X00218318 31939551

[38] Moraes ABT, Moulin MGB. Trabalho, vida e morte no setor de rochas ornamentais: efeitos psicossociais do acidente de trabalho fatal para a família. Cad. Psicol. Soc. Trab. [Internet]. 30º de junho de 2013 [citado 29º de outubro de 2024]; 16(1):25–40. Disponível em: https://www.revistas.usp.br/cpst/article/view/77740

